# Minimizing the Adverse Effects of Asymmetric Links: A Novel Cooperative Asynchronous MAC Protocol for Wireless Sensor Networks

**DOI:** 10.3390/s19102402

**Published:** 2019-05-26

**Authors:** Md. Mahedee Hasan, Amit Karmaker, Mohammad Shah Alam, Andrew Craig

**Affiliations:** 1 IICT, Bangladesh University of Engineering & Technology, Dhaka 1000, Bangladesh; mahedee.hasan@gmail.com (M.M.H.); amitkarmaker06@gmail.com (A.K.); 2 Department of Computer Science, Tennessee Technological University, Cookeville, TN 38505, USA; amcraig42@students.tntech.edu

**Keywords:** MAC, wireless sensor network, link asymmetry

## Abstract

As Wireless Sensor Networks (WSNs) grow in popularity, researchers are now focusing more on some challenging issues that significantly degrade overall performance, such as energy hole mitigation, link asymmetry minimization, etc. Link asymmetry is a problem that arises when the coverage distance between two adjacent nodes varies. It creates an obstacle to overcome when designing an efficient Medium Access Control (MAC) protocol for WSNs with low duty-cycling. This phenomenon poses an especially difficult challenge for receiver-initiated asynchronous MAC protocols, which are popular due to their relatively higher energy efficiency. Exploiting the benefits of cooperative communication has emerged as one of the viable solutions to overcome this limitation. Cooperative communication in WSNs has received a lot of attention in recent years. Many researchers have worked to create a MAC layer supporting cooperative communication. However, the association of cooperative communication with an asymmetric link is not studied in the literature. In this research work, COASYM-MAC, a cooperative asynchronous MAC protocol for WSNs, is proposed based on a receiver-initiated MAC protocol that uses the fact that nodes have alternate paths between them to reduce link asymmetry. A key feature of the proposed protocol is that the optimal helper node is selected automatically in case of link asymmetry. Simulations exhibited that COASYM-MAC performs significantly better than a state-of-the-art MAC protocol for WSNs that handles asymmetric links, ASYM-MAC.

## 1. Introduction

**Figure 1:**
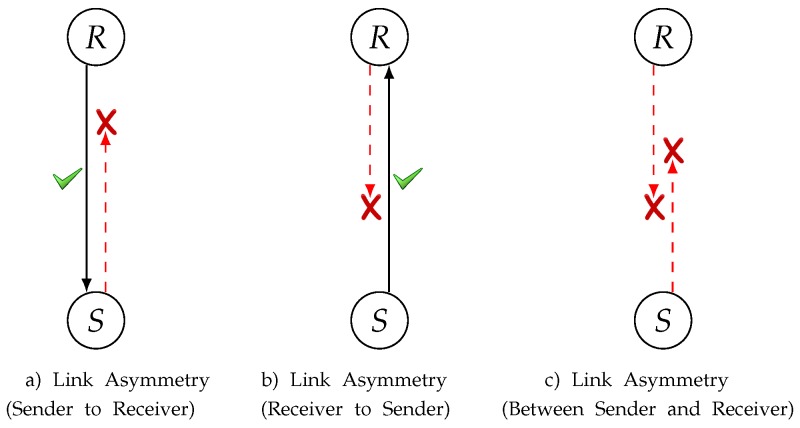
Asymmetric link situation.

Wireless Sensor Networks (WSNs) are one of the most promising technologies in decades. WSNs are frequently utilized in applications such as medical, autonomous vehicles, networking, smart homes, environmental areas and other emergency applications [1,2,3,4,5,6,7,8]. They are composed of huge quantities of sensor nodes capable of sensing, communication, processing, and storage [9,10]. Transferring sensing data between these nodes faces several challenges due to the node’s constraints, e.g., energy limitation [11], low computational capability, and low memory. Without the proper solution to these challenges, the current situation of WSNs cannot meet the requirements of Internet of Things (IoT) [12]. Since these nodes operate with little battery power, an important focus in research is making the sensor nodes live as long as possible. The majority of works, however, assume the link connecting two nodes to be fully symmetric. Asymmetric links, however, create possibilities where the same data is resent, wasting valuable energy. Link asymmetry is considered to be one of the most important characteristics of radio links, which can significantly affect the medium access layer’s performance. In low power WSNs, many of the wireless links are asymmetric [13]. Figure 1 gives scenarios where the links can be asymmetric. Figure 1a shows the scenario in which the sender is able to send data but the receiver cannot. In Figure 1b, the receiver is able to send the beacon and the sender cannot. Finally, in Figure 1c, neither side is able to send data and control information. In this research work, we consider a mixture of above three types whenever any link asymmetry occurs.

Several research works describe physical, logical, operational and legal reasons for link asymmetry [14,15]. Firstly, transmission range is the primary reasons for link asymmetry. D. Ganesan et al. found that the percentage of asymmetric links is negligible at short distances and increases significantly with higher distances [16]. Maximum transmission range of a sensor node is determined by the hardware properties such as the antenna and RF circuits. In these cases, transmission range problem cannot be solved without changing the nodes’ hardware such as the antenna or RF circuit. Secondly, link asymmetry may arise due to power limitation, which is a major challenge for WSN. It is highly likely that different nodes in WSN have different level of residual energy. For instance, Node *A* has enough energy to transmit data to Node *B*; however, Node *B* does not have enough energy to reach *A* or may choose not to reach Node *A* to save energy. Thus, non-uniform residual energy of sensor nodes is another major cause of link asymmetry. Thirdly, interference is another significant factor that causes asymmetric link. Suppose that Nodes *A* and *B* can reach each other, however, if Node *B* transmits at a power level that suffices to reach Node *A*, it may cause interfere with a Node *C* that is a valid user of the same frequency spectrum. This unintended situation occurs due to the co-existence of of different wireless technologies using a same frequency band. Thus, Node *B* is not allowed to transmit at a power level above a given threshold, which causes the link asymmetry. Finally, some environmental factors, obstacles, and even weather conditions may cause the link asymmetry [17]. In this paper, we only consider the varying transmission range of the sensor nodes as the cause of link asymmetry.

The link quality of wireless nodes can be measured by Link Quality Estimator (LQE). Two types of LQE are described in [18,19]: hardware-based and software-based. Received Signal Strength Indicator (RSSI) and Link Quality Indicator (LQI) are two types of hardware-based approaches. Sensor nodes provide a RSSI register that estimates the signal strength of the received packet. A LQI is another hardware-based vendor specific indicator where the first eight symbols are considered for scoring from 50 to 110. The higher is the value, the better is the link quality and vice versa. Packet Reception Ratio (PRR) is a software-based technique that measures link quality by calculating the proportion of packets that were successfully received to the number of packets transmitted in total. The absolute difference between PRR of sender to receiver and PRR of receiver to sender is used to identify the link asymmetry [20]. Several researchers analyze this difference to classify the wireless link. If this difference is greater than 1%, the link is asymmetric [21]. According to K. Srinibasan et al. [22], when the uplink and downlink PRR difference is greater than 40%, then the wireless link is considered asymmetric. L. Sang et al. transmitted 100 packets at different levels of power to analyze asymmetric links [13]. Table 1 shows that, at high power levels, links are mostly symmetric, but, as power level decreases, link asymmetry sees an increase.

Link asymmetry has significant impact on the quality of connectivity [23]. It degrades the overall network performance. In [24], P. Misra et al. showed that protocols that do not consider link asymmetry may work fine in simulation but fail completely in real life deployments. Hence, link asymmetry is one major but challenging issue in designing a MAC protocol for WSNs. A significant portion of radio links, observed in recent studies, are asymmetric in typical WSN deployment. It is also observed from test-bed results that asymmetric links mostly occur due to low transmission power. In such cases, nodes fail to transmit data which eventually make the network almost non-functioning. Therefore, considering link asymmetry while designing a MAC protocol is indispensable.

Battery power limitation is another key challenging issue in WSNs. “Synchronous duty cycling” is a preliminary approach to reduce energy consumption [25,26]. However, it needs clock synchronization, which increases the system overhead. To overcome this problem, “asynchronous duty cycling” approach is introduced that saves more energy compared to the synchronous approach. Asynchronous protocols work in two modes: sender initiated mode and receiver initiated mode. In sender initiated mode, sender sends preamble [27] and in receiver initiated mode, receiver sends beacon to seek attention of the transmitting node [28]. Previous finding shows that receiver initiated mode is more energy efficient than sender initiated mode [29]. Asynchronous MAC protocol in receiver initiated mode is therefore drawing much attention from the researcher community for its inherent capability of saving more energy. However, link asymmetry is a key factor that adversely affect receiver initiated asynchronous MAC protocols to a great extent where nodes mostly remain in sleep mode to save more energy. An extensive literature review presented in “Section 2: Related Work” shows that ASYM-MAC [30] works on an asynchronous mechanism that sufficiently takes the link asymmetry into consideration.

Nodes can keep a routing table that stores the link status of surrounding nodes. They can exchange a beacon message to find asymmetric links and update periodically. However, wireless sensor nodes are memory constrained. Moreover, extra periodic beacon exchange increases the possibility of collisions as well as energy consumption. To avoid the asymmetric link problem in low power asynchronous WSNs, we have been motivated to exploit cooperative communication without an extra control message exchange.

One of the apparent solutions to avoid the link asymmetry is maintaining and periodically updating the neighborhood table. Unlike our previous work reported in [31], here we consider a tree-based protocol. None of the existing work has used cooperative communication with a tree-topology with consideration to the asymmetric link situation to the best of our knowledge. In the preliminary version of this work, we avoid link asymmetry through cooperative communication, but we do not select an efficient cooperative node. In this work, however, we enlarge our earlier work in several aspects. This work’s specific contributions are as follow:

- We design a tree-based technique to solve link asymmetry between the sender to receiver, receiver to sender or both.
- In addition to the tree formation information, we develop an efficient algorithm to select an efficient helper node. Unlike ASYM-MAC, this scheme can deal with the asymmetric link scenario more strictly in low power WSNs.
- For effective helper selection, we propose a wake-up schedule algorithm for each node by which the most efficient node among sender’s neighbors wakes up early to send its beacon.
- The performance of the proposed protocol was evaluated through a simulation model based on the OMNeT++ simulation framework.
- Performance comparisons with state-of-the-art MAC protocol ASYM-MAC showed that the proposed protocol outperforms the ASYM-MAC protocol in terms of energy consumption, PRR, Packet Delivery Ratio (PDR), and end-to-end delay.

The organization of the remainder of this paper is as follows: In Section 2, the related works are elaborated. Section 3 discusses problem statements and network architectures. Section 4 presents COASYM-MAC, this paper’s proposed protocol. The experimental results are described in Section 5, and finally the conclusion and some future scope of this work are discussed in Section 6.

## 2. Related Work

Many researchers have proposed different MAC protocols in recent years with an aim to reduce the sensor nodes’ energy consumption. The most common method is duty cycling [25,26], which involves turning off the transceiver intermittently to reduce the amount of energy used. Next, a sender-initiated protocol is introduced [27] in which the sender sends a long preamble to seek the receiver’s attention. This method has advantages over the synchronous MAC protocol because nodes in sender-initiated protocols do not need clock synchronization to create a schedule. A long preamble does, however, cause additional overhead and introduce a higher chance of collision. Following this, the long preambles have been converted to packets. The most popular MAC protocol that packetizes the preamble is X-MAC [32]. X-MAC has shorter preambles instead of one long one, allowing the receiver to send acknowledgements early between the preambles. However, the shorter preambles still cannot completely overcome the extra overhead. Following sender-initiated protocols, the receiver-initiated protocol has been introduced [28]. In this protocol, the receiver initiates contact and sends a beacon to get the attention of the sender. This introduces significantly less overhead than the preambles sent in the sender-initiated protocols. However, our literature review presented in this section found that even state-of-the-art protocols have not taken link asymmetry into consideration, and therefore may not work properly whenever asymmetric link occurs in real life deployment. In ADP-MAC protocol [33], the receiver uses polling mechanism to ensure the reception of the preamble packet sent by the sender node. However, it does not consider the link asymmetry situation at any side. As a result, receiver fails to know the ongoing preamble packet by the sender in the case of sender to receiver link asymmetry and sender fails to get the early acknowledge by the receiver in case of receiver to sender link asymmetry. PW-MAC [34] works over RI-MAC, and it predicts the wake-up time of the receiver based on previous sending schedule of the intended receiver. It has reduced energy associated with the idle listening of sender node that avoids early wake up. However, if receiver to sender link asymmetry arises, the sender wakes up in time without finding any beacon from the receiver. In the case of link asymmetry, the PW-MAC completely fails to utilize the prediction of wake-up time. A-MAC [35], another receiver initiated asynchronous MAC protocol, works similar to RI-MAC but differs in the way that sender sends an auto acknowledgement message to the receiver to indicate that it has data to send. However, in the case of link asymmetry, receiver cannot receive data even though sender sends data packet after sending an acknowledgement. In EnRI-MAC [36], a notification message is sent by the sender before it receives beacon from the receiver. The goal of sending wake-up notification is to inform other senders within the range of the same intended receiver that it has data to send. Despite getting beacons from the intended receiver, other nodes refrain from sending data in the current cycle. In the case of link asymmetry, other senders cannot get the wake-up notification. In such case, multiple senders transmit their data to the receiver resulting in collision.

As already stated in Section 1, we explored the benefit of cooperative communication to avoid the asymmetric link problem. This section reviews related works divided into two broad categories: The asymmetric link based protocol followed by cooperative based protocol.

### 2.1. Asymmetric Link Based Protocol

Asymmetric links can drastically degrade the performance of low power WSNs. That is why some researchers have dealt with this asymmetric link in the network layer. P. Mitra et al. introduced Asymmetric Geographic Forwarding (A-GF) [37] where they discovered asymmetric links by broadcasting beacons in the network. They increased the stability of the asymmetry by maintaining neighbor tables. Finally, they proposed an efficient route for data delivery. AsymRp (Asymmetric convergecast Routing Protocol) was proposed by B. Romdhani et al. [38], who used two-hop neighbor information to select the common and intermediate neighbors for data and explicit acknowledgement transfer. However, using two-hop information in routing table increases the complexity. Moreover, due to memory constraint, holding a large number of information in table in a dense network has been a challenging task.

**Figure 2:**
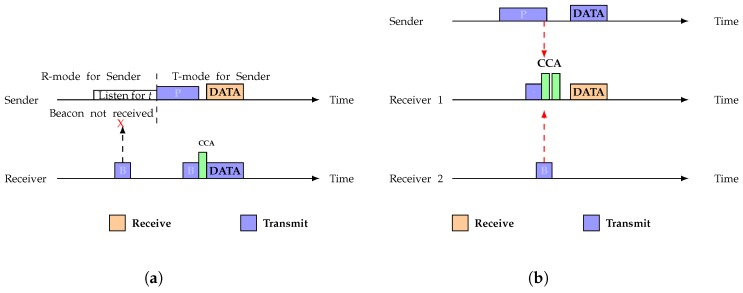
Working mechanism of ASYM-MAC protocol: (**a**) operation of the ASYM-MAC protocol; and (**b**) a collision scenario.

ASYM-MAC [30] works over RI-MAC considering duty cycling. ASYM-MAC combines sender and receiver initiated MAC protocol. By default, system operates on receiver initiated mode. When sender cannot get beacon from its receiver, it waits for `t’ time and then switches to sender initiated mode where it sends its data followed by a long preamble. However, when sender gets beacon from receiver but sender cannot send data due to link asymmetry, this protocol works very poorly. Figure 2a,b shows the working mechanism of ASYM-MAC. ASYM-MAC has two modes of operation: R-mode and T-mode. R-mode is the default mode in which a receiver-initiated MAC is used. ASYM-MAC switches its mode to T-mode when it fails to receive a probing packet from the intended receiver. This switching is triggered by a timeout that occurs after a certain period of time elapses without receiving the probing packet. In T-mode, the transmitter sends a series of small preamble packets to notify the receiver of the pending data transmission. Once the receiver captures the signal of on-going transmission of preamble packets, it also receives data from the sender.

In [39], K. Pengwon et al. tried to solve the asymmetric link problem by using LQI and a dual tree topology. They used link quality estimators to construct the dual tree, where one is a collection tree and another is a dissemination tree. ERPPro [40] performs energy-efficient reverse path routing for an asymmetric link. This protocol uses residual energy and link quality information to select a legitimate reverse path. L. Vyas et al. proposed a Dynamic Hierarchical Agglomerative Clustering-based protocol by considering asymmetric links [41]. Besides WSNs, some other researchers [42,43,44] have dealt with asymmetric links in underwater sensor networks and wireless mesh networks. L. Shu et al. observed the link asymmetry problem in duty cycled Industrial Wireless Sensor Networks [45]. They showed that, when the link asymmetry increases, the nodes sleep less, hence, lifetime decreases.

### 2.2. Cooperative Based Protocol

In this paper, we deal with asymmetric links. Moreover, we exploit the cooperative communication in asymmetric links. There are many cooperative-based protocols in wireless sensor networks. Among the various types of cooperative based protocol, some of them consider asynchronous communication. In this work, we also consider the asynchronous mechanism, thus, in this subsection, we discuss some prominent asynchronous cooperative based communication mechanisms.

A.B. Nacef et al. proposed CL-MAC [46], which uses cooperative communication in low power sensor networks. In CL-MAC, a piece of a shorter preamble packet is sent to each of the sender’s relay and destination nodes. This small preamble notifies all relays and receivers to awaken at a universal rendezvous (RDV) point. The main disadvantage of CL-MAC is the need for time synchronization, and the preamble, even though it is small, creates additional overhead. ARQCRI [47] works over CL-MAC and tries to disregard time synchronization. When receivers send beacons, the sender transmits a coop message to notify the receiver to wait a few moments for the selection of the relay. When the relay is selected, then the sender transmits the data to the receiver and the relay. This method successfully ignores time synchronization, but is unable to handle the situation in which the sender is unable to send the coop message. CPS-MAC [48] uses a series of short preambles to seek the attention of the receiver and all the neighbor nodes, which creates extra overhead like in CL-MAC. It offers a tree-based mechanism with one sibling, but, if there is more than one sibling, then there may be a possibility to relay data to more than one neighbor, which may create a collision. There is no mechanism for effective relay selection. In RIC-MAC [49], there is no need for an extra preamble to wake up the receiver and neighbor nodes at a common RDV Point, as it uses two different wake-up beacons for the receiver and relay. The main disadvantage is that it creates huge idle listening. Moreover, similar to CPS-MAC and ARQ-CRI, it does not offer effective relay selection. Table 2 shows the comparative analysis of some prominent asynchronous cooperative MACs for WSNs. Upon finding their inappropriateness, this paper attempts to build a cooperative communication based protocol that not only avoids the asymmetric link situation, but also selects relay nodes efficiently without the extra control message exchange or other clock synchronization problems. Thus, in short, a protocol is designed to avoid the link asymmetry situation.

## 3. Problem Statement

As previously discussed, in ASYM-MAC, nodes default to receiver-initiated mode. If the sender is unable to obtain a beacon from the intended receiver because of an asymmetric link, it will switch modes to be sender-initiated and send a preamble to the receiver, after which the data can be sent. There are some drawbacks to this protocol.

### 3.1. Preamble and Data Collision

**Figure 3:**
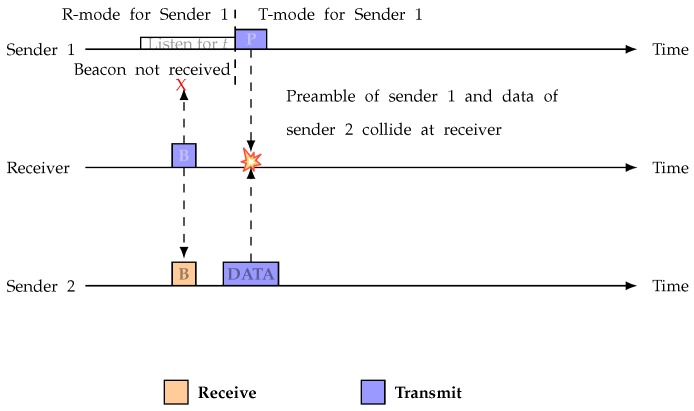
Preamble and data collision.

A collision can occur between the data and the preamble when nodes suddenly switch into sender-initiated mode. When a beacon is sent to Sender 1 and Sender 2 by Receiver 1, Sender 2 is able to find the beacon, but Sender 1 does not find the beacon because of an asymmetric link. Sender 1 will then go into sender-initiated mode and begin transmitting the preamble. Figure 3 shows how this can collide with the data being sent by Sender 2 at Receiver 1’s end. A. Pegatoquet et al. showed that, when the network has more than 15 nodes, the ASYM-MAC protocol introduces highest data collision rate [51]. However, in our proposed protocol, there is no transition between receiver initiated and sender initiated mode. Therefore, this type of collision never occurs in our proposed protocol.

### 3.2. No Mechanism in Data Loss Situation

**Figure 4:**
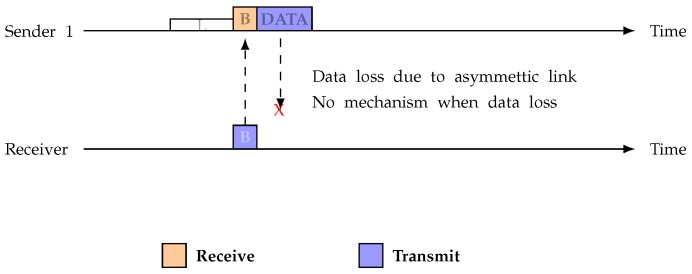
Data loss due to link asymmetry.

Since link asymmetry can happen at either end, an asymmetric link can be found where Sender 1 tries to transmit data to the receiver. ASYM-MAC does not have a method stating how the sender can transmit in this situation. Figure 4 describes the problem in the data loss situation. Our protocol considers that the link asymmetry can happen at any end. Thus, our protocol can handle the data loss situation.

### 3.3. Control Packet Overhead

When nodes switch into sender-initiated mode, they send long preambles which creates additional overhead. We avoid the packetized preamble to manage the link asymmetry situation.

## 4. The COASYM-MAC Protocol

As already stated, we consider a tree formation that can select relay nodes within a sibling to avoid the asymmetric link problem. We divide this section into four subsections: system model and network topology, tree formation, wake up scheduling, and data collection.

### 4.1. System Model and Network Topology

Let N nodes be randomly deployed over a certain area by G=(V,E), where *V* represents the set of all vertices (*V* = $v_{1}$, $v_{2}$ … $v_{n}$) and *E* is the set of edges which represents communication links between nodes. *S* represents the Base Station (BS). The BS is placed on one side of the sensor node. The wireless links are not symmetric, which is the main assumption of this paper. The summary of the used notation is listed in Table 3.

### 4.2. Tree Formation

In WSNs, nodes are deployed at random in the monitoring region and report the gathered data to the BS in a converge-cast (many to one) manner [52]. In this subsection, we discuss tree formation topology in detail. Tree formation can be divided into three stages. In the first stage, the distance of each node from the Base Station is calculated and one-hop count nodes are selected. In the second stage, each node selects a temporary parent under various conditions; however, there may be a child node imbalance among parent nodes. Hence, we balance the number of child nodes in the third stage.

#### 4.2.1. Identifying First Hop and Distance for Each Node

During this stage, the BS sends a “HELLO” message to each node. Every node calculates how far it is from the base station ($d_{i,BS}$) [53]. To make our protocol more realistic, we did not consider GPS availability. Each node sends a firsthopcount request within its range $r_{i}$. The nodes that receive the acknowledgement from the Base Station are considered as first hop nodes.

#### 4.2.2. Parent Selection

In this section, the first hop node sends a requestforwarding message to its neighbor. The message contains <Node id, $d_{BS}$, hop count>. Following this request message, each node temporarily selects its parent node using Algorithm 1.

Each node checks all its neighbors to see whether the node can select it as a parent node. When Node *u* first finds a request forwarding message, it selects itself as the parent node. Then, it continues checking further neighbor requests as per Algorithm 1. Finally, Node *u* selects a temporary parent node.

**Algorithm 1** Tree Formation.
1:$P_{u}$ = ∅                           ▹ initially parent is empty2:$Q_{u}$ = ∅                      ▹ initially buffer for parent is empty3:**for** each Node $i\in N_{u}$**do**          ▹$N_{u}$ is the set of all neighbor nodes of u in G4:    **if** $P_{u}$ is empty **then**5:        **if** $d_{i,BS}$ <$d_{u,BS}$ **then**6:           $P_{u}$=*i*                      ▹ Assign current node as parent7:           add *i* in $Q_{u}$8:        **end if**9:    **else**10:        **if** ($d_{i,BS}$ <$d_{u,BS}$ && $d_{i,u}$ <$d_{u,P_{u}}$) && $hcount_{i,BS}$≤$hcount_{P_{u},BS}$ **then**11:           $P_{u}$=*i*12:           add *i* in $Q_{u}$             ▹ Add current node into the parent queue13:        **end if**14:    **end if**15:
**end for**
16:Sort $Q_{u}$ according to distance and hop count17:**for**∀$Q_{u}$ each element in buffer **do**18:    $P_{u}$ = Dequeue($Q_{u}$)19:    Ack ←**GetAcknowledgement($P_{u}$)**20:    **if** (Ack ≠ Overflow) **then**21:        Exit from loop and $P_{u}$ is the parent of *u*22:    **end if**23:
**end for**



#### 4.2.3. Balanced Tree Formation

Node *v* receives the parent request from several neighbor nodes. However, if it allows all requesting nodes, then there may be possibilities to create an imbalanced tree. To overcome this limitation, Node *v* can allow a certain number of nodes, e.g., $P_{TH}$. Line 19 in Algorithm 1 gets the value of Ack from Algorithm 2.

**Algorithm 2** GetAcknowledgement($P_{u}$).
1:$NoC_{v}$ = ϕ             ▹ initially number of child of Node *v* ($NoC_{v}$) is empty2:**if** (count($NoC_{v}$) ≥$P_{TH}$) **then**3:    Ack ←Overflow4:
**else**
5:    Add in $NoC_{v}$                 ▹ Add $P_{u}$ into the number of child set6:    Ack ←True7:
**end if**
8:return Ack


### 4.3. Wake Up Scheduling

Duty cycling is the best technique for saving a significant amount of energy. A. Fujimoto et al. proposed a depth based wake up scheduling protocol in [54] based on the node’s depth without considering various factor like residual energy, distance, etc. In this section, we introduce multi-factor based wake up scheduling so that an ordinary node can select efficient relay nodes for data forwarding. At first, in this tree structure, we calculate the duty cycle time of each node by multiplying fixed wake up times by the tree level δ

(1) Duty Cycle=δ×Fixed Wakeup time

As discussed in Section 4.2.2, each node knows its parent node. Now, we calculate the factor value of each node using the following equation:

(2) $$Factor\;value=\alpha \times CSI+\beta \times RE+\gamma \times \frac{1}{d_{u,p_{u}}}$$

Here, RE is the residual energy, and $d_{u,p_{u}}$ is the distance between a node and its parent. We consider three factors to calculate the factor value of each node. A node measures the Channel State Information (CSI) by using the Received Signal Strength Indicator (RSSI) [55]. To establish a relationship among channel state information (CSI), residual energy (RE) and the distance, three constants, α, β and γ, are incorporated. From the simulation results, we found that a value of 0.4 for α, 0.4 for β and 0.2 for γ gives the optimum result for calculating factor value. Now, we calculate the backoff score from Equation (3). Here, the node that has a comparatively high CSI, high residual energy, and low parent child distance takes the short backoff.

(3) $$backoff=\frac{1}{Factor\;value}$$

Now, each node wakes up periodically by combining the duty cycle time with the backoff time using Equations (1) and (3).

(4) Node Waking Time=Duty Cycle+Backoff

Thus, nodes under the same parent as well as the same level will wake at the same time with Equation (1), but they differ in the backoff time. In the traditional MAC protocols, backoff is used for delaying the opportunity of transmission by a sender when multiple senders are contending for data transmission, thereby reducing the probability of collision. In such cases, transmission time is closely related to the backoff. However, in our proposed protocol, we use backoff to achieve a different goal. Here, backoff is used to differentiate the wake-up schedule among all siblings so that the node having the highest factor value (Equation (2)) wakes up early (Equations (3) and (4)) to send the beacon first. Unlike CL-MAC, here we consider distance because distance is one of the main factors for choosing the best node as a relay node. Since wake-up schedule of nodes are different from neighboring nodes, there is less probability of collision by hidden terminal problem. We formally represent the details of the wake-up schedule algorithm in Algorithm 3.

**Algorithm 3** Wake up scheduling.
1:**for** each node in the network **do**2:    Determine the CSI by using RSSI, residual energy and distance from the parent node;3:    Calculate the factor value according to Equation (2) by using the information from Step 2;4:    Compute the backoff value according to Equation (3);5:    Compute the duty cycle according to Equation (1);6:    Determine the node wake-up time according to Equation (4);7:
**end for**



### 4.4. Data Collection

This section briefly discusses the data collection strategy. We consider both situations when the link is symmetric and when the link is asymmetric.

#### 4.4.1. In Case of Symmetric Link

**Figure 5:**
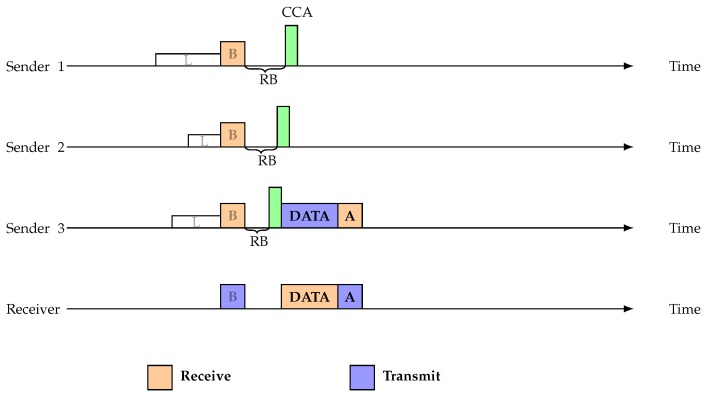
Symmetric link situation.

Upon waking up, the parent node transmits beacons to each of its children. The child, which is waiting to send data, pauses for a random short backoff and performs a Clear Channel Assessment (CCA). If the CCA finds that the channel is available, the child will transmit the data. If the CCA finds that the channel is unavailable, the child will wait for the parent to send a new beacon. In Figure 5, each of the three children are trying to transmit data and are listening to the channel. The receiver transmits the beacon when it wakes up. Once the children receive the beacon, they each backoff for a random time to prevent collisions. Sender 3 chooses the lowest backoff time and wins control of the channel. It sends its data and stands by for an acknowledgement from the receiver.

Algorithm 4 presents the pseudo-code that summarize the operation when link is symmetric.

**Algorithm 4** Link symmetric.
1:**if** a beacon packet received from $P_{u}$ **then**          ▹ Child *u* sending data to Parent $P_{u}$2:    Child takes a random backoff and checks the CCA3:    **if** win the channel **then**4:        send data to $P_{u}$ and go to sleep mode5:    **else**6:        wait for next beacon7:    **end if**8:
**end if**
9:**if** a data packet received from $P_{u}$ **then**         ▹ receiver $P_{u}$ received data from child *u*10:    send ACK to sender11:
**end if**



#### 4.4.2. In Case of Beacon Loss Due to Link Asymmetry

If a child (*u*) is ready to send data, it will hold until the parent ($P_{u}$) sends it the beacon. The sender will wait for *t* time, where *t* is chosen to be double a node’s wakeup time. If the child is unable to receive the beacon from its parent because of link asymmetry in under *t* time, the child will use a cooperative node for data forwarding. The cooperative node may be the sibling of the child node or any other neighbor node with equal or lower hop count than the child node. In dense networks, keeping all neighbor node information is very difficult because of memory constraints. Thus, in this paper, we do not use the neighbor table. We only include parent ids and hop counts in the beacon message. By using the beacon information, the child node can make the decision of data forwarding. Now, after exceeding *t* time, the child *u* waits for the beacon of its sibling. The sibling that has a comparatively high CSI, high RE, and low distance from parent first sends the beacon as per Equation (4). After getting the beacon child *u* immediately sends the data. Other children of the parent $P_{u}$ take the Short Random Backoff (SRB) and when it checks the channel it finds the ongoing transmission, as described in Algorithm 5. In the tree topology, any parent can receive messages other than its child. When children send data packets to siblings, they add parent IDs to the data packets.

**Figure 6:**
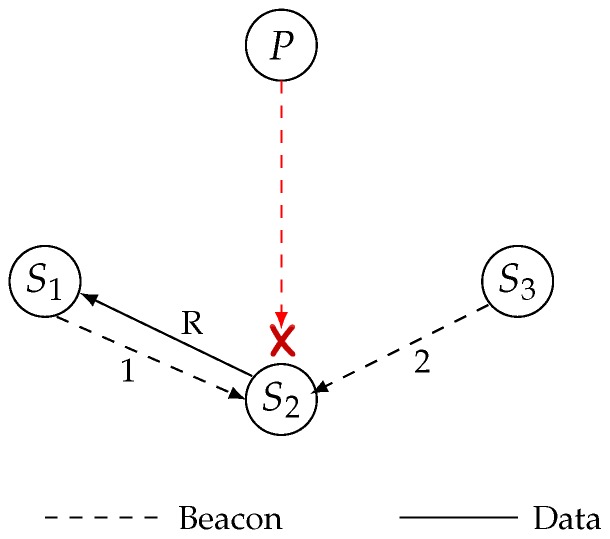
Relay selection among sibling.

**Figure 7:**
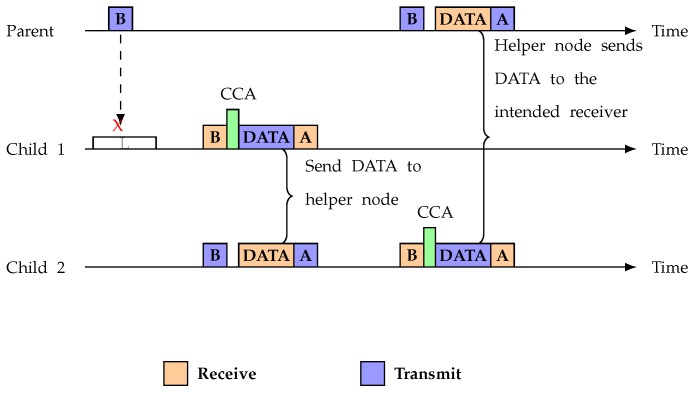
Beacon loss situation.

Conversely, if a child Node *u* does not get a beacon from any of its siblings within another *t* time, then it waits for the beacon from any of its neighbor that have an equal or lower hop count to the Base Station. If the child gets the beacon from any of its neighbors who satisfy the condition, then it immediately sends the data packet with identifying relay mark so that the neighbor node does not discard the data packet. Figure 6 shows the relay selection among siblings and Figure 7 displays the timing diagram of the proposed protocol in the case of beacon loss.

#### 4.4.3. In Case of Data Loss Due to Link Asymmetry

**Figure 8:**
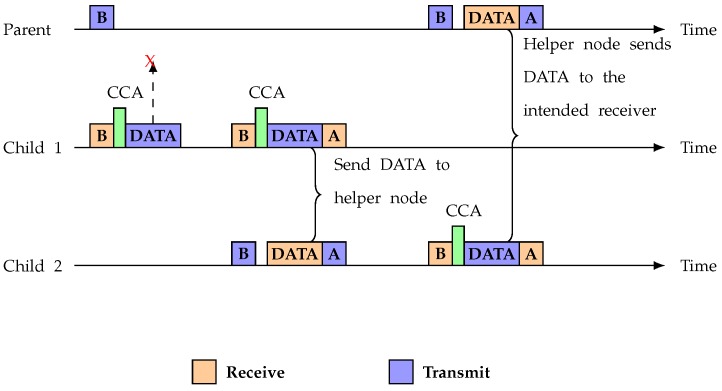
Data loss situation.

Link asymmetry can sometimes also happen from child *u* to parent $P_{u}$. Child *u* is waiting for the parent’s beacon to send its data. Once it successfully wins the channel through random backoff, child *u* sends its data but parent $P_{u}$ cannot receive the data because of an asymmetric link. The sender will assume it is an asymmetric link because it will wait *t* time (half of the beacon interval) but cannot get an ACK from the receiver. Child *u* will then choose a cooperative node, as described in Algorithm 5. Figure 8 displays the timing diagram for the scenario where data are lost because of link asymmetry.

Algorithm 5 presents pseudo-code to summarize selecting a relay in the case of beacon loss (receiver-to-sender asymmetry) and data loss (sender-to-receiver asymmetry).

**Algorithm 5** Cooperative communication.
1:Child *u* sending data to Parent $P_{u}$2:**if** Beacon or ACK not received from $P_{u}$ within *t* **then**3:    sender waits for the beacon from its sibling4:
**end if**
5:**if** Child *u* receives a beacon from its sibling *s* **then**6:    sends its data packet with relay indicator7:
**else**
8:    Child *u* waits for another *t* time9:    **if** child gets beacon from neighbor *n* **then**10:        check the hopcount from beacon11:        **if** $hopcount_{n1}$ ≤ $hopcount_{u}$ **then**12:           sends data to neighbor *n*13:           change parent of *u* to *n*14:        **else**15:           waits for beacon from another neighbor *n*16:        **end if**17:    **end if**18:
**end if**



## 5. Simulation Results

### 5.1. Simulation Environment

We evaluated and compared our protocol’s performance with a recognized asymmetry-based MAC protocol ASYM-MAC with simulations done using OMNeT++. The metrics evaluated were the average energy consumption in the network, in addition to energy consumption per node, average delay per packet, Packet Reception Ratio (PRR), Packet Delivery Ratio (PDR), System Throughput, and Control packet overhead with link asymmetry. The network simulated was made up of of a sink node and either 27 or 54 nodes for two scenarios in a 1000 × 1000 m${}^{2}$ region. Both protocols ran under the same parameters. The parameters are listed in Table 4. The data generation time was distributed uniformly between 0.01 and 0.99 s. In this research work, we considered all the applications that acquire data periodically, usually for early detection of natural calamities such as earthquakes, volcanic eruptions, land slides, etc. [56,57,58], therefore have a uniform distribution of inter-arrival time. Such applications require continuous monitoring of the environment, and generate packets at a fixed interval. Furthermore, sensor nodes used in some IoT based applications also use such uniform distribution [59]. The time needed to transition from receiver-initiated mode to sender-initiated mode was 1 s using ASYM-MAC, which is equal to the waiting time (*t*) of the proposed protocol.

### 5.2. Simulation Results

#### 5.2.1. Energy Consumption Results

**Figure 9:**
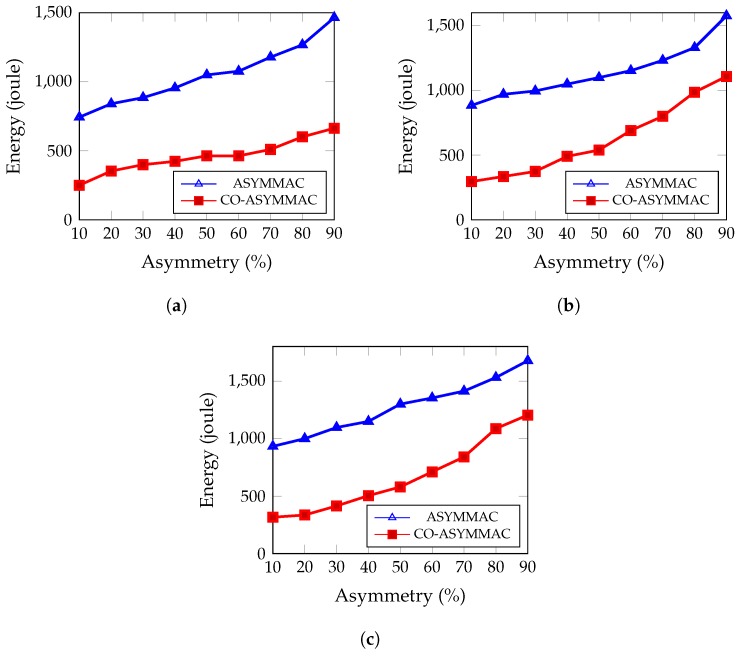
Average energy consumption: (**a**) for 27 nodes; (**b**) for 54 nodes; and (**c**) for 90 nodes.

**Figure 10:**
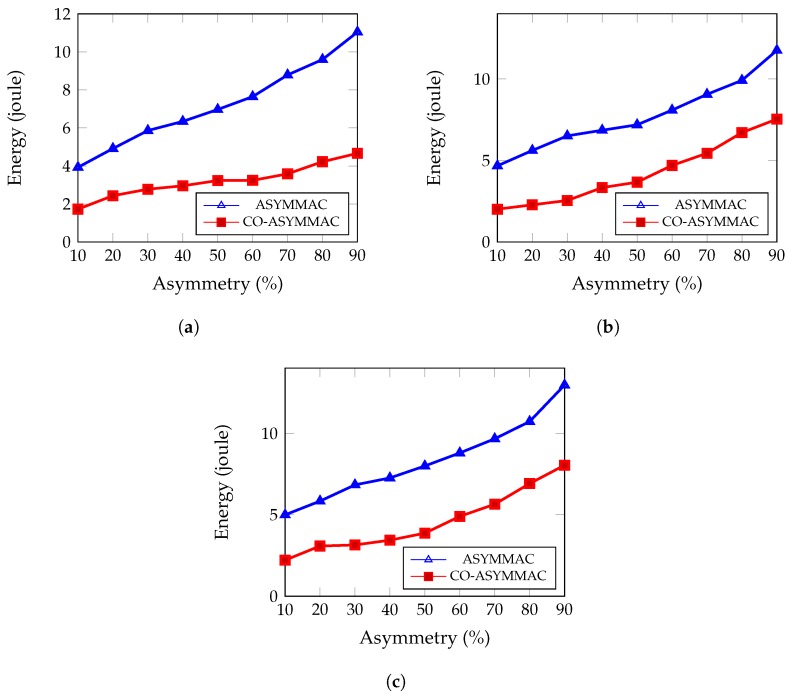
Per packet energy consumption: (**a**) for 27 nodes; (**b**) for 54 nodes; and (**c**) for 90 nodes.

Energy is one of the key challenges of WSNs. Substantial energy loss happens due to link asymmetry. Figure 9 and Figure 10 show the network’s average energy consumption and energy consumption per packet, respectively.

Average energy consumption of the network is calculated as total amount energy used (control packet, data packet, idle listening) by all members of the network divided by the total number of nodes. Here, the proportion of asymmetric links was varied to populate simulation results. Conversely, per packet energy consumption is defined as the energy consumption in the network to the number of successfully transmitted packets.

(5) $$E_{avg}=\frac{E_{DATA}+E_{cp}+E_{idl}}{N}$$

Here, $E_{avg}$ represents average energy consumption per node, $E_{DATA}$ is energy consumption for transmitting data, $E_{cp}$ represents energy consumption for the control packet, $E_{idl}$ represents energy consumption for idle listening, and N represents the network’s total number of nodes.

Figure 9 exhibits the relationship between percentage of asymmetric links and average energy consumption. It was observed that, as asymmetric links increase, the node’s power consumption increases as well. In ASYM-MAC, it was identified that if a node switches into sender-initiated mode and transmits the preamble, nearby nodes are not aware of the change. As a result, there are many collisions between the beacon and the preamble, adding to energy consumption. The preamble also creates extra overhead, which consumes more energy. In the case of 50% link asymmetry, the percentage of average energy consumption for COASYM-MAC protocol is reduced by 56% for 27 nodes (Figure 9a), by 51% for 54 nodes (Figure 9b), and by 55% for 90 nodes (Figure 9c) compared to ASYM-MAC protocol.

(6) $$E_{packet}=\frac{E_{consumption}}{\mathrm{Total}\;\mathrm{generated}\;\mathrm{data}\;\mathrm{packet}}$$

Here, $E_{packet}$ is the Energy consumption per packet and $E_{consumption}$ is the total energy consumption in the network.

Figure 10 shows that, with the increase of link asymmetry, energy per packet is also increased. COASYM-MAC can effectively reduce this increase in energy consumption. In the case of 50% link asymmetry, it can reduce the energy consumption per packet by 54% for 27 nodes (Figure 10a), by 49% for 54 nodes (Figure 10b), and by 51% for 90 nodes (Figure 10c) as compared to the ASYM-MAC protocol.

#### 5.2.2. Packet Reception Ratio

**Figure 11:**
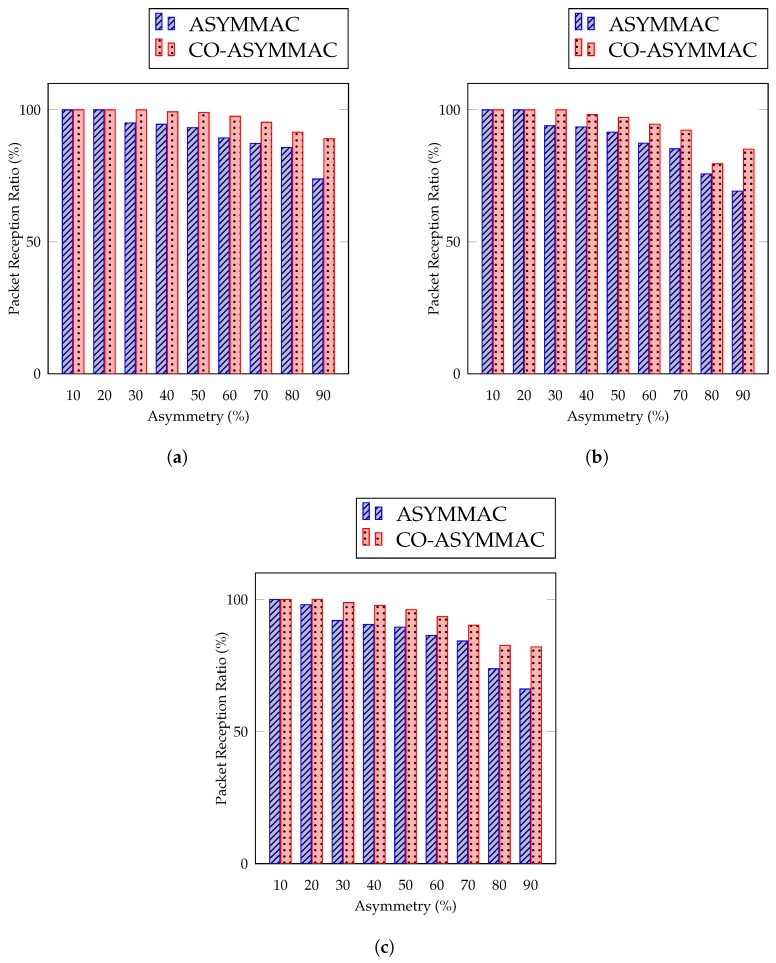
Packet reception ratio: (**a**) for 27 nodes; (**b**) for 54 nodes; and (**c**) for 90 nodes.

Packet reception ratio is calculated as the percentage of total packets received to total packets transmitted. Figure 11 compares packet reception ratio (PRR) with a different number of asymmetric links. The figure shows that, if the number of asymmetric links is below 30%, the PRRs of both protocols remain the same. However, if there is an increase in the number of asymmetric links, the PRR curve of ASYM-MAC declines much faster than COASYM-MAC.

(7) $$PRR=\frac{\mathrm{Number}\;\mathrm{of}\;\mathrm{received}\;\mathrm{packets}}{\mathrm{Number}\;\mathrm{of}\;\mathrm{transmitted}\;\mathrm{packets}}$$

It was also observed that, if the link asymmetry occurs from sender to receiver, then the PRRs of both protocols remain approximately the same; however, if the link asymmetry occurs from either receiver to sender or on both ends, then COASYM-MAC’s PRR is far better than ASYM-MAC. The PRR was also compared with the existing ASYM-MAC protocol with varying asymmetric nodes. The PRR of COASYM-MAC is better than ASYM-MAC because, in ASYM-MAC, the receiver cannot receive data in the case of link asymmetry. On the other hand, COASYM-MAC overcomes this problem by using a cooperative communication technique. The sender waits *t* time and then sends a long preamble. After getting the receiver’s attention, it sends the data to the receiver. In this long time period, the receiver receives few packets compared to packets transmitted. The data reception rate decreases with the increase of asymmetric percentages. This is true for ASYM-MAC as well as COASYM-MAC, but, in the case of link asymmetry for COASYM-MAC, where the sender cannot transmit data to the receiver, it waits *t* time and sends data using cooperative nodes. In the case of 90% of link asymmetry, COASYM-MAC performs better than the ASYM-MAC in terms of PRR as it achieves 17% improvement for 27 nodes (Figure 11a), 19% for 54 nodes (Figure 11b), and 20% for 90 nodes (Figure 11c).

#### 5.2.3. Packet Delivery Ratio

PDR is calculated as the percentage of packets successfully transmitted to packets generated.

(8) $$PDR=\frac{\mathrm{Number}\;\mathrm{of}\;\mathrm{received}\;\mathrm{packet}}{\mathrm{Number}\;\mathrm{of}\;\mathrm{generated}\;\mathrm{packet}}$$

**Figure 12:**
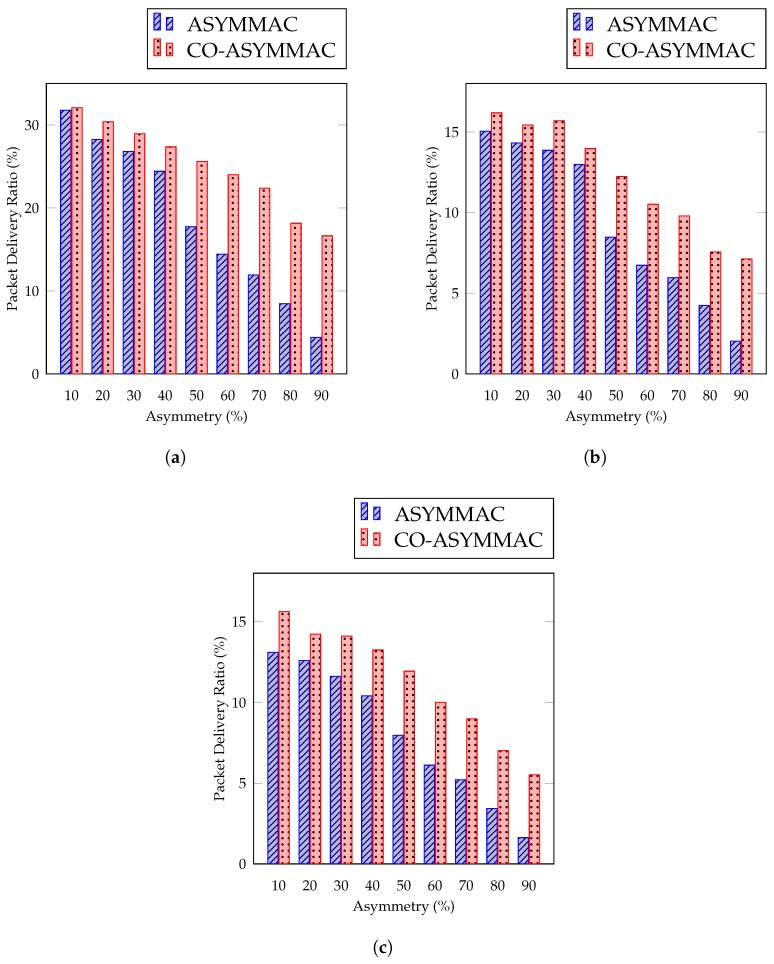
Packet delivery ratio: (**a**) for 27 nodes; (**b**) for 54 nodes; and (**c**) for 90 nodes.

PDR over link asymmetry is illustrated in Figure 12. The proposed protocol improves the packet delivery ratio by approximately 10% considering asymmetric percentage from 10% to 90%. The PDR of COASYM-MAC is higher than ASYM-MAC because, in ASYM-MAC, the sender cannot send data to the receiver; it waits *t* time and then sends the long preamble in the case of link asymmetry. In that duration, the new packets are generated to the sender; this increases the generated packets rather than transmitted packets and decreases the PDR. On the other hand, in COASYM-MAC, if the sender cannot transmit data to the receiver, it waits *t* time and sends data using cooperative nodes. If the sender cannot get the beacon from its siblings, it waits another *t* time for a neighbor’s beacon. If it gets the beacon from a neighbor, it immediately makes this neighbor a parent and solves link asymmetry permanently. As a result, PDR is increased in COASYM-MAC over ASYM-MAC. The percentage of gain in packet delivery ratio for COASYM-MAC is 74.2%, 71.4%, and 70.47% for 27 nodes (Figure 12a), 54 nodes (Figure 12b), and 90 nodes (Figure 12c), respectively, as compared to the ASYM-MAC protocol when link asymmetry is 90%.

#### 5.2.4. Average Delay per Packet

**Figure 13:**
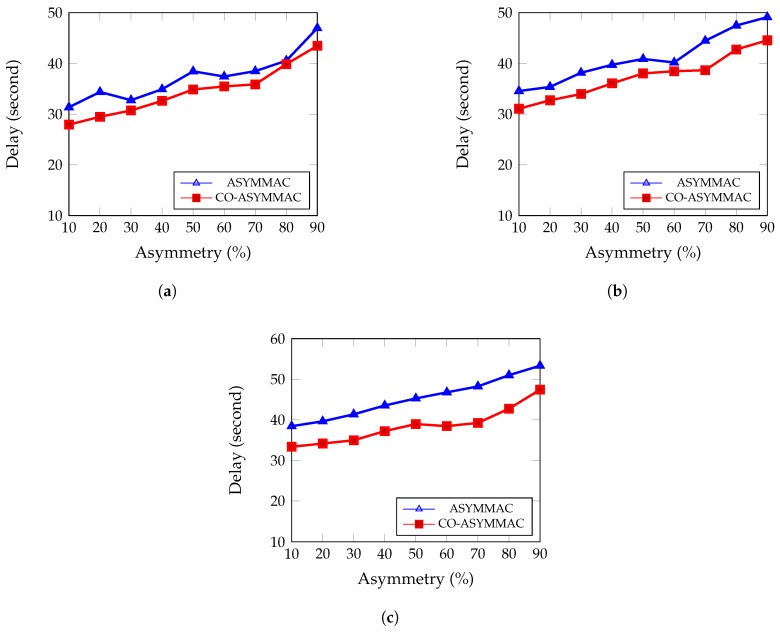
Average delay per packet: (**a**) for 27 nodes; (**b**) for 54 nodes; and (**c**) for 90 nodes.

For an urgent monitoring application, the delay can be a crucial parameter. Average delay per packet can be calculated as the sum of the duration of time that all packets take to reach the Base Station (BS) divided by the total number of packets that successfully reach the Base Station. It is also called end-to-end delay. Figure 13 displays the relationship between average delay per packet and the number of asymmetric links.

(9) $$AverageDelay=\frac{\sum \mathrm{Time}\;\mathrm{to}\;\mathrm{send}\;\mathrm{all}\;\mathrm{data}\;\mathrm{in}\;\mathrm{BS}}{\mathrm{Number}\;\mathrm{of}\;\mathrm{data}\;\mathrm{successfully}\;\mathrm{reached}\;\mathrm{at}\;\mathrm{BS}}$$

Figure 13 shows that in COASYM-MAC the average delay is decreased more than 5% compared to ASYM-MAC. When link asymmetry surpasses 80%, most nodes must select a relay node to send data to the intended receiver. The COASYM-MAC protocol gives better performance compared to ASYM-MAC. In ASYM-MAC, if a sender fails to transmit a packet due to an asymmetric link, every time it waits for *t* time, switches to sender-initiated mode, and sends a long preamble each time. In the COASYM-MAC protocol, however, when link asymmetry occurs it waits for *t* time and chooses a sibling. If it does not find any sibling as helper node, it waits for a neighbor node’s beacon. This neighbor node sends data to the BS as a helper node. The sender node immediately changes its parent to the neighbor node. As a result, the average delay decreases in COASYM-MAC compared to ASYM-MAC. The percentage of reduction in average delay per packet for COASYM-MAC in the case of 90% link asymmetry is 7.45%, 9.28%, and 11.08% for 27 nodes (Figure 13a), for 54 nodes (Figure 13b), and for 90 nodes (Figure 13c), respectively, as compared to the ASYM-MAC protocol.

#### 5.2.5. System Throughput

**Figure 14:**
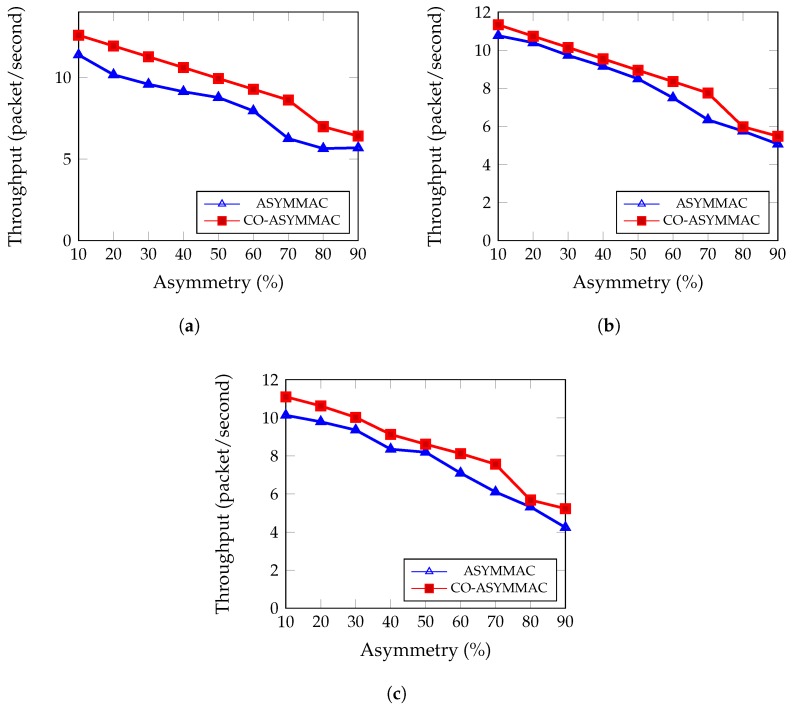
System Throughput: (**a**) for 27 nodes; (**b**) for 54 nodes; and (**c**) for 90 nodes.

System throughput is defined as the total number of data packets delivered from the source to the destination within a certain period of time. Figure 14 shows the throughput comparison between two MAC protocols with the percentage of link asymmetry. The proposed protocol improved the system throughput by 15% when the network link asymmetry is 10%. However, in 90% link asymmetry, the proposed protocol improved the system throughput by almost 10%. For 54 nodes (Figure 14b), however, the overall throughput improved by 10%. Considering all situations, COASYM-MAC improves performance over ASYM-MAC. In case of link asymmetry, COASYM-MAC uses a cooperative node to send data or changes its parents rather than sending a control packet If a node changes its parent in run time due to link asymmetry, it solves the link asymmetry problem permanently for the next time. Since it sends data without using any control packets, it increases throughput. Figure 14c shows the results of same metrics for 90 nodes which agree with the previous characteristics.

(10) $$System\;Throughput=\frac{\mathrm{Number}\;\mathrm{of}\;\mathrm{packet}\;\mathrm{received}}{\mathrm{Overall}\;\mathrm{network}\;\mathrm{operation}\;\mathrm{time}}$$

#### 5.2.6. Control Packet Overhead

**Figure 15:**
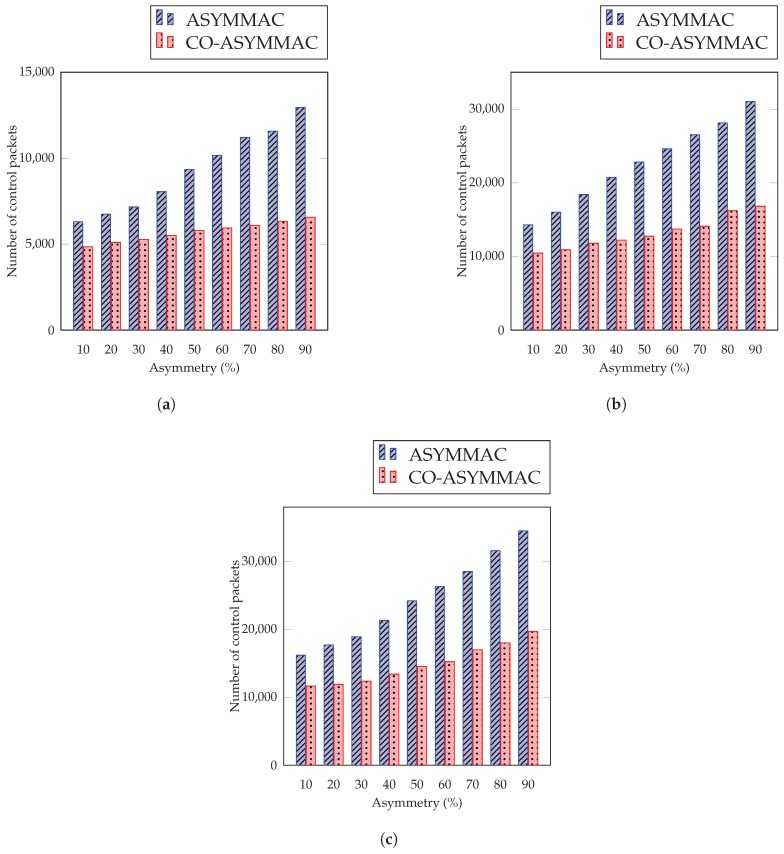
Control Packet Overhead (**a**) for 27 nodes (**b**) for 54 nodes (**c**) for 90 nodes.

In the effective relay selection, the COASYM-MAC reduces the control packet overhead of the network as shown in Figure 15, and also immensely decreases the control packet transmission of the overall network. In ASYM-MAC, when the node performs a transition from the sender-initiated mode to receiver-initiated, it sends a burst preamble to seek the attention of the receiver and it happens every time when the sender wants to send data. Whereas in COASYM-MAC, the nodes wait for the beacon of a sibling to perform the cooperative communication. If it does not get a beacon from its sibling, then it waits for a neighbor beacon whose distance or hop count is lower than this node. Then, it sends data via this cooperative node and makes this node a parent permanently. Thus, it does not need to use a control packet for the next time. As shown in Figure 15, COASYM-MAC decreases control packet overhead by 48.26%, 46.84%, and 42.23% for 27 nodes, (Figure 15c), for 54 nodes (Figure 15b), and for 90 nodes (Figure 15c) respectively as compared to the ASYM-MAC protocol.

## 6. Conclusions

Link asymmetry poses a major challenge for many wireless sensor network based applications that require high reliability as well as stringent QoS parameters. While asynchronous MAC protocol provides significant improvement in energy efficiency, it very poorly deals with the link asymmetry. In this paper, a novel cooperative communication based asynchronous medium access control protocol (COASYM-MAC) is proposed for minimizing the adverse effects of asymmetric links and fulfilling sufficient QoS requirements. It forms a tree architecture among the nodes that allows a node to choose an alternative path to the sink in case of link asymmetry. Such tree formation significantly decreases the hidden terminal collisions. It also proposes a wakeup scheduling algorithm that uses the level number, duty cycle, and metrics value (residual energy, channel state information and distance) for the dynamic selection of wakeup time for each node. Finally, an efficient relay node selection algorithm is proposed that takes into consideration both cases: when the link is fully symmetric and when there is an asymmetric link. OMNeT++ based simulation was carried out to compare the performance of COASYM-MAC protocol with the state-of-the-art ASYM-MAC protocol. Results show that the proposed protocol outperformed the existing ASYM-MAC protocol in terms of energy efficiency, throughput, delay, and control packet overhead.

## Figures and Tables

**Table 1:** Link asymmetry in different power level [13].

Tx Power	Symmetric	Asymmetric	Unidirectional
	(<10%)	(10–90%)	(>90%)
−19 dBm	50%	43%	7%
−14 dBm	65%	22%	13%
−5 dBm	88%	6%	6%

**Table 2:** Comparative analysis of different asynchronous Cooperative MAC.

MAC	Extra	Clock	Idle	Reliability	Relay
	Preamble	Sync	Listening		
Cl-MAC [46]	Yes	Yes	Less	No	Yes
CPS- MAC [48]	Yes	Yes	Less	Yes	No
ARQ-CRI [47]	No	No	Huge	No	No
ACT-MAC [50]	Yes	No	Huge	Yes	No
RIC- MAC [49]	No	No	Huge	No	No

**Table 3:** Summary of the used notation.

Symbol	Description
G(V,E)	Graph of vertex *V* and edge *E*
$Ne_{u}$	Neighbor of a Node *u*
$P_{u}$	Parent of Node *u*
$d_{u,BS}$	Distance from Node *u* to Base Station
$hcount_{u,BS}$	Number of hop from Node *u* to Base Station
$RE_{u}$	Residual energy of Node *u*
$r_{u}$	Transferring radius of Node *u*
$PReq_{v}$	Parent request for Node *v*

**Table 4:** Simulation parameters.

Parameters	Values
Node deployment area	1000 m × 1000 m
Number of nodes	27/54/90
Node’s initial energy	2000 J
Simulation Time	150 s
Sensing Range	70 m
$T_{x}$ Energy	0.50 J
$R_{x}$ Energy	0.25 J
Asymmetry (%)	10 ∼ 90
ACK Timeout	0.4 s
Queue Length	256 Packet
Waiting Time *t*	1 s
